# Osteoporosis Recognition in Rats under Low-Power Lens Based on Convexity Optimization Feature Fusion

**DOI:** 10.1038/s41598-019-47281-7

**Published:** 2019-07-29

**Authors:** Jie Cai, Wen-guang He, Long Wang, Ke Zhou, Tian-xiu Wu

**Affiliations:** 1 School of Information Engineering, Guangdong Medical University, Zhanjiang, 524023 China; 2 School of Basic Medical Science, Guangdong Medical University, Zhanjiang, 524023 China

**Keywords:** Image processing, Osteopetrosis

## Abstract

Considering the poor medical conditions in some regions of China, this paper attempts to develop a simple and easy way to extract and process the bone features of blurry medical images and improve the diagnosis accuracy of osteoporosis as much as possible. After reviewing the previous studies on osteoporosis, especially those focusing on texture analysis, a convexity optimization model was proposed based on intra-class dispersion, which combines texture features and shape features. Experimental results show that the proposed model boasts a larger application scope than Lasso, a popular feature selection method that only supports generalized linear models. The research findings ensure the accuracy of osteoporosis diagnosis and enjoy good potentials for clinical application.

## Introduction

According to the World Health Organization (WHO), osteoporosis is a systemic skeletal disease where reduced bone mass and deteriorated microarchitecture increase the bone fragility and the risk of a broken bone^1,2^. This definition attaches equal importance to the mass and quality of bones. The bone quality is demonstrated by the microarchitecture, i.e. the 3D structure and connections of trabeculae. The variation in trabecular microarchitecture is a major determinant of bone fragility. In the diagnosis of osteoporosis, it is even more sensitive than the popular index of bone mineral density (BMD)^3,4^. Therefore, the trabecular microarchitecture must be included in the research of osteoporosis.

Osteoporosis is now a common health problem among senior citizens in both developed countries like the UK and developing countries like China. In the UK, about 536,000 new fragility fractures occur each year^5^, leading to severe pain and disability to individual sufferers. In 2010 alone, osteoporosis treatment costed the National Health Service (NHS) of over £4.4 billion. Population ageing has become a serious problem in China. In 2017, the number of people over 60 in China had exceeded 240 million, about 17.3% of the total population. Among them, 158 million were aged 65 or above, accounting for 11.4% of the total population^6^. Many of these elderlies are faced with osteoporosis. Studies have shown that 33% of males and 73.8% of females aged between 70 and 79 in China are suffering from this disease. Against this backdrop, it is imperative to realize early warning, diagnosis and effective intervention of osteoporosis.

The existing studies on osteoporosis mainly relies on bone texture analysis, a non-invasive method that provides rich information about bones, using images produced by such imaging techniques (e.g. X-ray, microscopy, micro computed tomography (micro CT) and magnetic resonance imaging (MRI)). For example, Yang Xinhui *et al*.^7^ extracted the trabecular textures from X-ray images on bones, classified ten X-ray images on bones by machine, and proved the application potential of texture analysis. R. Karunanithi *et al*.^8^ analysed the trabecular features in X-ray images on the calcaneus of postmenopausal women, and discussed the causes of osteoporosis through the analysis. F. Mallard *et al*.^9^ performed texture analysis on the X-ray images of spine, distal radius and calcaneus, realized the early detection of changes in the microarchitecture of bones, and demonstrated the values of texture analysis for preventing and treating osteoporosis. Muthu Subash Kavitha *et al*.^10^ conducted a texture analysis on the X-ray images of the teeth, revealing that texture analysis, coupled with conventional indices, can greatly facilitate the diagnosis of osteoporosis. Using X-ray images and CT images, Lespessailles, Chappard, Castellanos *et al*.^11,12^ demonstrated the excellent effect of texture analysis in determining the fragility, mass and microarchitecture of bones, and the capacity of texture analysis in the diagnosis and early detection of clinical osteoporosis. Ji Ming and Chen *et al*.^13,14^ extracted the texture parameters of trabeculae from MRI images, and found significant texture differences between osteoporotic group (OVX group) and sham-operated group (SHAM group). The above studies confirmed the feasibility of texture analysis in osteoporosis research.

Recent years saw the application of many classification systems in the identification and classification of osteoporosis, such as multi-layer perceptron^15^, Bayesian classifier^16^, random forest classifier^17^ and support vector machine (SVM)^10,18^. The recognition accuracy of these systems hinges on the resolution of the source images. For instance, Frighetto^19^ obtained shape and texture parameters from spinal MRI images, and probed deep into the effect of the k-nearest neighbours (KNN) algorithm. Lately, the popular fuzzy logic has been proved efficient in osteoporosis diagnosis. Muthu Subash Kavitha^20^ developed a way to diagnose osteoporosis based on the hybrid genetic swarm fuzzy (GSF) classifier. Similarly, the grey level co-occurrence matrix (GLCM) have been employed for texture recognition of osteoporosis. As a popular approach for texture feature analysis, the GLCM can describe the spatial distribution of different levels in a image using a second-order statistic^21–23^.

Deep learning has achieved great success in the field of computer vision processing and has been applied to the medical field, especially to deal with image recognition and classification^24,25^, lesion segmentation, computer-aided diagnosis^26,27^ and microscopic image analysis^28^. Profound learning algorithm plays a vital role in assisting doctors to diagnose accurately and efficiently and becomes a supplement to human skills. Its models include supervised learning methods and unsupervised learning methods. Supervised learning methods need to give sample labels in advance. Classification models are trained by labeled image features. Classification categories are usually specified in advance. (1) Supervised learning methods are generally been adopted to support Vector Machines(SVM), Convolutional Neural Networks (CNN), Recurrent Neural Networks a (aRNN)and so on.Convolution network has successfully reduced the dimension of image recognition problem with huge amount of data through a series of methods, and finally it can be couched. Cem M. Deniz *et al*. presented an automatic proximal femur segmentation method that is based on deep convolutional neural networks (CNNs)^29^. CNNs have the unique capability of feature learning,it learn increasingly complex features from data automatically^30^. RNN is a kind of neural network that can predict the future to some extent while being used to analyze time series data (such as analyzing stock prices, predicting buying and selling points). Qiuling Suo, *et al*. proposed a multi-task framework that can monitor the multiple status ofdiagnoses,Patients’ historical records were directly fed into a Recurrent Neural Network (RNN) which memorizes all the past visit information, and then a task-specific layer is trained to predict multiple diagnose^31^. (2) Unsupervised learning method automatically classifies different classes according to the similarity between samples without preset sample labels.Such Encoders as Autoencoder(AE), Stacked Autoencoder (SAE), Staked Sparse Autoencoder(SSAE), deep belief network(DBN), Deep Boltzmann Machines(DBM), Variational Auto-Encoder(VAE)are all in common use. Among them, AE is used for dimension reduction or feature learning. Sparse Automatic Coding (SSAE) is a special three-layer neural network with sparse constraints in general neural networks whose model aims to obtain a low-dimensional representation from the original data. SSAE can automatically learn features from unlabeled data and can give better feature descriptions than the original data^32,33^. When it comes to practical application, sparse auto-coding will function as the preprocessing process of data. Stack self-coding neural network (SAE) is used as a neural network composed of multi-layer sparse self-encoders, and the output of the former layer self-encoder is used as the input of the latter layer. The Stack-type neural network parameters can be achieved through greedy couching layer by layer. Yassine Nasser, *et al*. proposed a novel aided diagnosis method for osteoporosis consisting of SSAE and a SVM classifier^34^. Changhang Xu, *et al*. introduced a deep learning algorithm—stacked autoencoder (SAE) to enhance the visibility of delaminations^35^ DBN and DBM are deep learning models belonging to the boltzmann family wihch use restricted boltzmann machine (RBM) as the learning module. The Restricted Boltzmann Machine (RBM) is a generative stochastic neural network. DBNs have undirected connections at the top two layers which form an RBM and directed connections to the lower layers. DBMs have undirected connections between all layers of the network^36^.

Deep learning skill is not the ultimate algorithm but represents several personalized schools in artificial intelligence, and its performance limit needs to be reasonably evaluated. In-depth learning, especially supervised learning, relies too much on high-quality, labeled big data. If there is a lack of high-quality labeled training samples. The trained model may be over-fitted or has poor robustness, so the obtained model needs to be tested for universality and applicability in various situations, which may not be suitable for the economic effect of medical images. Unsupervised learning has no such restriction and can work without supervision,embodying DBNs/DBMs. However, it is difficult to accurately estimate the joint probability and calculate the cost when creating DBN, which constitutes a defect. On the other hand, if automatic encoders generate errors in the first layer, they are likely to become invalid, which is one of their shortcomings. Such errors may cause network learning to reconstruct the average value of training data. At the same time, the research of in-depth learning in the field of Medical Image Computing is still mainly based on technology, and the evaluation index adopted is also an evaluation index in the field of computers. For any medical application, we would like to see the evaluation of related technologies according to medical rules. For example, multi-center, randomized and controlled research proves that this technology has more significant advantages than previous technologies.

In our previous research, the trabecular texture of rat tibial slices was investigated under the microscope. It is found that the ovariectomized group (OVX group) differed greatly in trabecular texture from the sham-operated group (SHAM group)^37–41^. In addition, the images on rat tibial slices were classified and recognized under 4 × and 10 × microscopes. The recognition rate was always above 90%. Compared to 2.5 × microscope, 4 × and 10 × microscopes can acquire the texture clearly. However, neither of the two microscopes can provide the whole picture of a slice. The only alternative is to take multiple images of the slice by manual positioning. Thus, it is difficult to maintain the morphological integrity of the trabeculae. What is worse, the experimental results may be affected by the inevitable overlapping or missing of images and the variation in light sources. By contrast, the 2.5 × microscope can capture a complete rat tibial slice, but the resulting image is too unclear to be recognized with common texture parameters. To solve these problems, this paper weighs the texture parameters considering the impacts from direction and distance, carries out feature fusion with shape parameters, and then recognizes the osteoporosis in the SHAM group and the OVX group.

Meanwhile, the previous research only extracts some of the texture parameters. With only a few parameters, there is no feature redundancy and no need for feature selection. In this paper, however, a total of 28 texture and shape parameters are extracted. In view of the strong correlation between these parameters, it is necessary to eliminate feature redundancy and find out effective feature parameters for the diagnosis of osteoporosis. Considering the poor medical conditions in some regions of China, this paper attempts to develop a simple and easy way to extract and process the bone features of blurry medical images and improve the diagnosis accuracy of osteoporosis as much as possible. Therefore, a convexity optimization model was proposed based on intra-class dispersion, which is capable of producing parameters suitable for classification and recognition. The proposed model boasts a larger application scope than Lasso, a popular feature selection method that only supports generalized linear models. The research lays a solid diagnostic basis for osteoporosis with poor equipment conditions and unclear image features.

## Methodology

### Experimental animals and feeding conditions

Three-month-old female Sprague Dawley (SD) rats with a mean weight of 250.1 g were selected for our experiment. These rats were divided randomly into the SHAM group and the OVX group. In the SHAM group, the rats were sham-operated and administered with 5 mL/kg·d of normal saline through the gastrointestinal tract. In the OVX group, the rats were ovariectomized and administered with 5 mL/kg·d of normal saline through the gastrointestinal tract. Each group has 10 rats and the experiment lasts 90 days.

The feeding conditions are as follows: Keep indoor temperature at 24~28 °C and control humidity between 50% and 60%. Keep the rats in separate cages in a special room, two in each cage. Change the mats every other day. Provide distilled water to the rats and let them drink the water freely. Feed them with the standard feed provided by the animal centre of our hospital. Weigh the rats once a week.

### Sample processing and preparation

All rats were injected with 25 mg/kg tetracycline hydrochloride into the subcutis once 13 days and 14 days before being killed (the first fluorescent labelling), and with 5 mg/kg calcein into the subcutis once 3 days and 4 days before being killed. The two fluorescent labels are separated by 10 days.

At the end of the experiment, the rats were anaesthetized by intraperitoneal injection with 3% amytal sodium (1.5 mL/kg), and then killed by drawing the all the blood from right ventricle. This is to reduce the red blood cells in the marrow, which may otherwise disturb the observation and analysis of bone slices.

The upper segment of the tibia was extracted and made into non-decalcified bone slices. Each sample was cut into thin slices (5μm thick) and thick slices (9 μm thick). The thin slices were strained with toluidine blue, while the thick ones were directly mounted. The static parameters of the slices were detected by bone histomorphometry and calculated by relevant formulas.

### Devices and software

The main devices include tungsten carbide steel knife (Leica, Germany), RM2155 hard tissue microtome (Leica, Germany) and automated image digitization analysers (e.g. light microscope & fluorescence microscope) (Nikon, Japan). The bone histomorphometry measurement software (KSS Scientific Consultants, US) and MaZda texture analysis software were adopted for result analysis.

### Feature extraction

A total of 52 valid images of rat tibial slices were obtained for our experiment. Among them, 34 belong to the SHAM group and 18 belong to the OVX group. All of the images were taken under a 2.5 × microscope.

### Extraction of trabecular texture parameters

**Figure 1:**
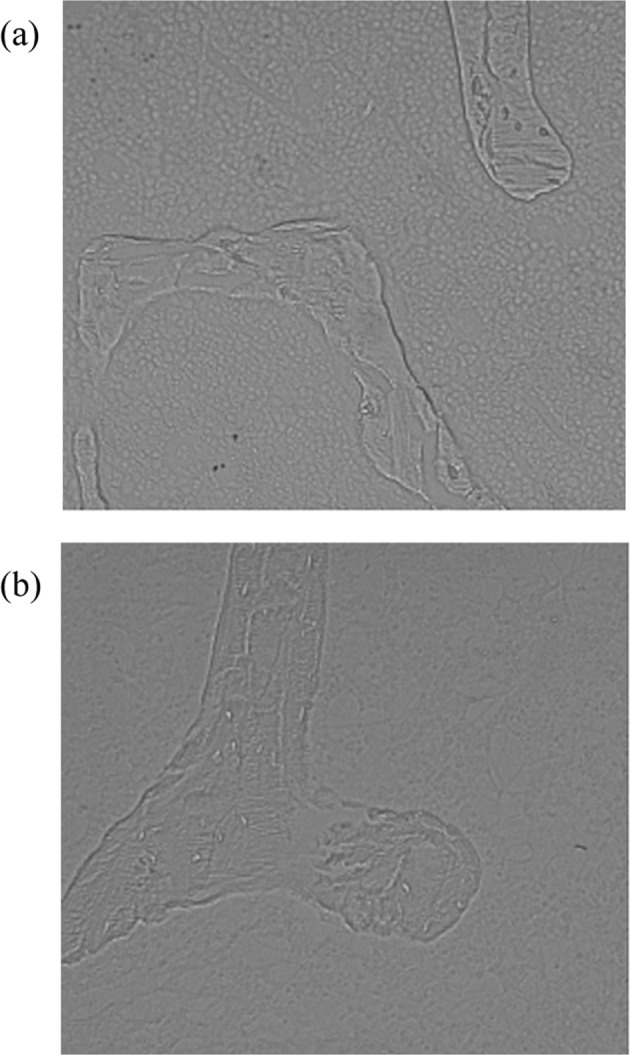
Trabecular images, (**a**)SHAM group; (**b**) OVX group.

Based on previous studies, we found that there was a significant difference in trabecular bone texture between OVX group and SHAM group under high power microscope, as shown in Fig. 1, so it is important to identify the texture of trabecular microarchitecture from the images.Here, a total of 16 texture parameters are extracted, including 11 from the GLCM and 4 from the grey-level run length matrix (GLRLM). The 11 parameters extracted from the GLCM are angular second moment, contrast, correlation, variance, sum of variance, mean sum, entropy, sum entropy, entropy difference, residual variance and inverse difference moment. The 4 parameters extracted from the GLRLM are run-length non-uniformity, grey-level non-uniformity, short run emphasis and long run emphasis.

### Extraction of shape parameters

**Figure 2:**
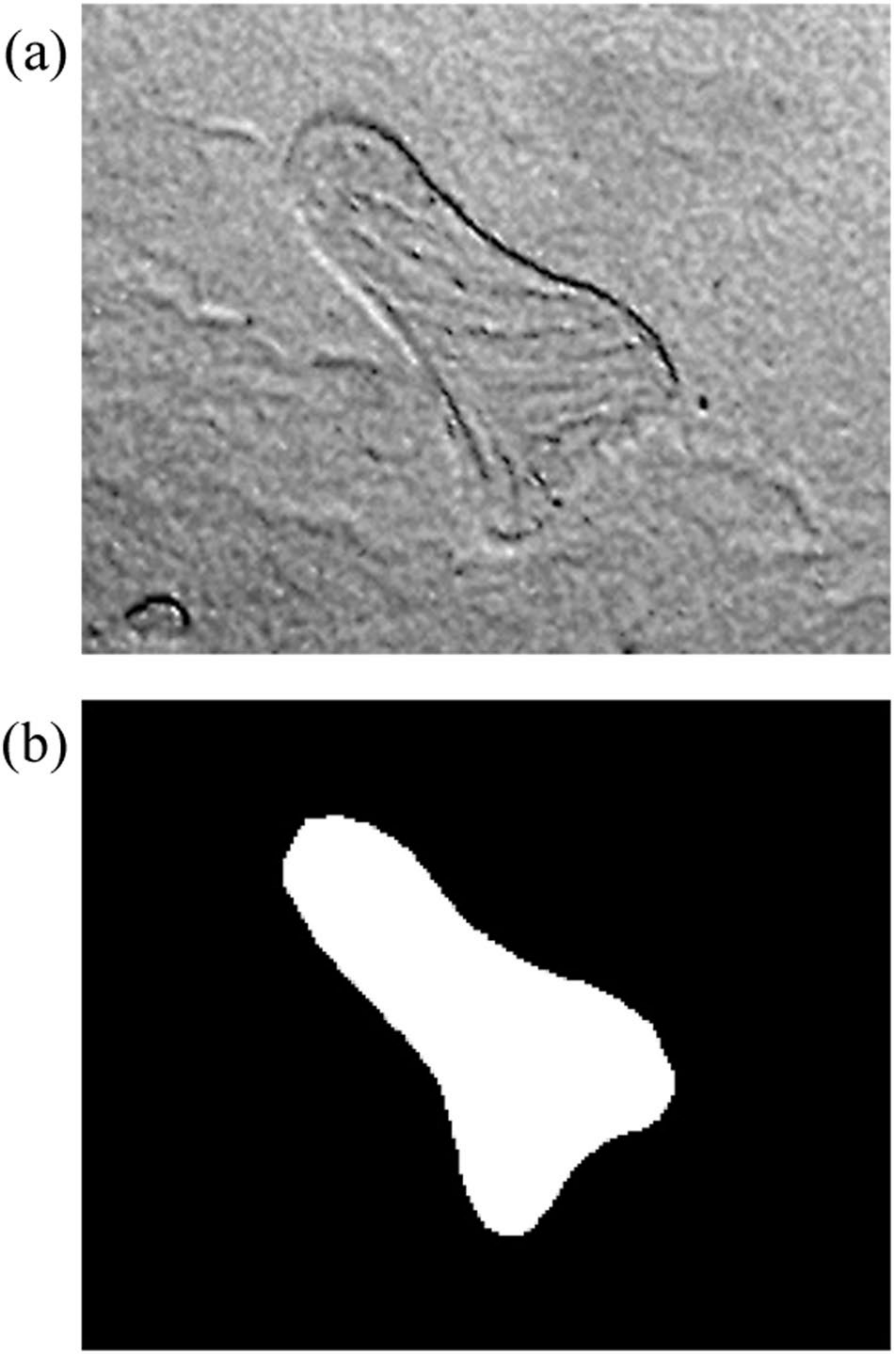
Delineation of the region of interests (ROI), (**a**) Trabecula; (**b**) ROI.

As mentioned before, the 2.5× microscope can capture a complete rat tibial slice, but the resulting image is too unclear to be recognized with common texture parameters. Besides, shape is an important feature of trabecular microarchitecture. Hence, the shape features of trabeculae were extracted and combined with the texture feature parameters, aiming to enhance the recognition accuracy. Figure 2 presents the extracted trabecular shapes.

Based on the extracted shapes, some shape parameters of the ROI were calculated and 6 dimensionless shape parameters were selected as follows:

Solidity = Area/Convex surface area

Regional density = Area/Perimeter^2^

Correction rate = Incircle diameter/Excircle diameter

Convexity = Convex surface perimeter/Perimeter

Roundness = Area/Maximum diameter^2^

Length-width ratio = Length of minimum bounding rectangle/Width of minimum bounding rectangle.

In addition, 7 Hu’s invariant moments were extracted to reflect the geometric features of the ROI. These parameters are so named because they are invariants with respect to translation, scale and rotation. In image processing, geometric invariant moments can be treated as an important feature of the object and a basis for image classification. Thus, a total of 13 shape parameters are extracted in this study.

### Data processing

It is well known that four directions, namely 0°, 45°, 90° and 135°, must be considered during the extraction of texture parameters from the GLCM and the GLRLM. Hence, a feature parameter has four values, each of which corresponds to one of the four directions. What is more, the texture parameters extracted from the GLCM are also influenced by distance. However, the impacts of direction and distance on texture parameters are often overlooked in the existing studies. The traditional approach is to set the distance at any direction to 1 or the mean value of the distances at all four directions.

Considering the limited number of pixels in the trabecular image under a low-power lens, the GLCM may be affected greatly by any slight variation in the grey-levels of adjacent pixels. As a result, the extracted texture parameters must be further processed before use. Here, the texture parameters are weighed by variation coefficient method, with the most direction-sensitive and distance-sensitive parameters serving as weight coefficients

### Weighted texture parameters

The parameters should be given different weights in different directions. The variation coefficient method was adopted to calculate the weighting coefficient, due to its abilities to highlight the variation in each direction and discriminate between different values. Specifically, the most direction-sensitive GLCM texture parameters were identified and used to calculate the weighting coefficient. The algorithm can be described as follows.

Let *x* (*i*, 1), *x* (*i*, 2), *x* (*i*, 3) and *x* (*i*, 4) be the values of texture parameters of the *i-*th sample image, which are the most sensitive to the directions of 0°, 45°, 90° and 135°, respectively. Then, the mean value of these direction-sensitive parameters can be expressed as:

$$\overline{{x}_{i}}=\frac{1}{4}\sum _{j=1}^{4}x(i,j)$$

The variation coefficient in each direction is:

$$\begin{array}{cc}v(i,j)=\frac{|x(i,j)-\overline{{x}_{i}}|}{\overline{{x}_{i}}} & (j=1,2,3,4)\end{array}$$

The weighting coefficient in each direction is:

$$\begin{array}{cc}w(i,j)=\frac{v(i,j)}{\sum _{j=1}^{4}v(i,j)} & (j=1,2,3,4)\end{array}$$

In this way, we have the weight of each sample in all four directions. Let *p* (*i*, *j*) (*j* = 1, 2, 3, 4) be the values of a texture parameter of the *i*-th sample image in each direction. Then, the final value of this texture parameter of the *i*-th sample image can be expressed as:

$$\overline{{p}_{i}}=\sum _{j=1}^{4}w(i,j)p(i,j)$$

Similarly, the most direction-sensitive GLRLM texture parameters were identified and used to calculate the weighting coefficient. The specific process is the same as the above.

### Influence of distance on texture parameters

As mentioned before, the GLCM may be affected greatly by any slight variation in the grey-levels of adjacent pixels, due to the limited number of pixels in the trabecular image under a low-power lens. Therefore, the distance selection directly bears on the results of image analysis. The optimal experimental effect hinges on the selection of proper point-to-point distance. In this paper, the texture parameters under the most stable distance are selected after analysing the variation in parameter value with distances.

### Feature selection based on a convex optimization model

#### Matrix representation of intra-class dispersion

Let matrices A_1_(A_1_ $\in \,{R}^{{n}_{1}\times p}$) and A_2_ (A_2_ $\in \,{R}^{{n}_{2}\times p}$) be the first-type and second-type samples in the sample space A, respectively. Then, the intra-class dispersion can be expressed as:

$$S={S}_{1}+{S}_{2}=\sum _{i=1}^{2}||{A}_{i\ominus }\overline{{A}_{i}}|{|}_{F}^{2}$$

where Θ is element subtraction; $\overline{{A}_{i}}$ is the mean value of the samples in class i, which is a p-dimensional vector.

Let ${C}_{i}={A}_{i\ominus }\overline{{A}_{i}}$. Then, the intra-class dispersion can be expressed as:

$$S={S}_{1}+{S}_{2}=||{C}_{1}|{|}_{F}^{2}+||{C}_{2}|{|}_{F}^{2}$$

#### Intra-class dispersion representation after feature selection and mapping

Let matrices A_1_(A_1_ $\in {R}^{{n}_{1}\times p}$) and A_2_ (A_2_ $\in {R}^{{n}_{2}\times p}$) be the first-type and second-type samples in the sample space A, respectively. If p features ${a}_{1},{a}_{2},\cdots ,{a}_{p}$ are extracted from each matrix, then the two sample data can be expressed as:

$$\begin{array}{ccc}(\begin{array}{cccc}{a}_{11}^{A1} & {a}_{12}^{A1} & \cdots  & {a}_{1p}^{A1}\\ {a}_{21}^{A1} & {a}_{22}^{A1} & \cdots  & {a}_{2p}^{A1}\\ \cdots  & \cdots  & \cdots  & \cdots \\ {a}_{{n}_{1}1}^{A1} & {a}_{{n}_{1}2}^{A1} & \cdots  & {a}_{{n}_{1}p}^{A1}\end{array}) & and & (\begin{array}{cccc}{a}_{11}^{A2} & {a}_{12}^{A2} & \cdots  & {a}_{1p}^{A2}\\ {a}_{21}^{A2} & {a}_{22}^{A2} & \cdots  & {a}_{2p}^{A2}\\ \cdots  & \cdots  & \cdots  & \cdots \\ {a}_{{n}_{2}1}^{A2} & {a}_{{n}_{2}2}^{A2} & \cdots  & {a}_{{n}_{2}p}^{A2}\end{array})\end{array}$$

Suppose there exists a linear function (projection plane) such that the projections of A_1_ and A_2_ onto it satisfy the condition: the distance between all projections in the same sample is minimized. Let b = (b1, b2, …, bp) T be the mapping coefficient vector. Then, the linear function can be expressed as follows:

$$y(A)={b}_{1}{a}_{1}+{b}_{2}{a}_{2}+\cdots +{b}_{p}{a}_{p}$$

Then according to the matrix representation in 3.3.1, the intra-class dispersion after mapping shall be expressed as:

$${S}_{w}^{b}={S}_{1}^{b}+{S}_{2}^{b}=||{C}_{1}\circ b|{|}_{F}^{2}+||{C}_{2}\circ b|{|}_{F}^{2}$$

where “o” is the element-wise product operation.

#### Convex optimization method

The following convex optimization problem is considered:

$$\mathop{{\rm{\min }}}\limits_{b}||Ab-Y|{|}_{2}^{2}+\frac{||{C}_{1}\circ b|{|}_{F}^{2}}{{n}_{1}}+\frac{||{C}_{2}\circ b|{|}_{F}^{2}}{{n}_{2}}+{\lambda }_{1}||b|{|}_{1}\,({\rm{P}})$$

The loss function is generally expressed as a typical Lasso model: $||{\rm{Ab}}-{\rm{Y}}|{|}_{2}^{2}$. In many cases, however, feature selection should facilitate the subsequent classification and recognition. Therefore, the intra-class dispersion after selection and mapping must be minimized, in addition to ensuring the closeness of the selected features with the predicted variables. Thus, it is necessary to consider the P-shape optimization problem, whose objective function is $f(b)=||Ab-Y|{|}_{2}^{2}+\frac{||{C}_{1}\circ b|{|}_{F}^{2}}{{n}_{1}}+\frac{||{C}_{2}\circ b|{|}_{F}^{2}}{{n}_{2}}$ and penalty function is $pt(b)={\lambda }_{1}||b|{|}_{1}$_._

**Lemma 1**. The $f:\,{R}^{P}\to R$ is a C_1_ smooth convex function, whose gradient ∇f satisfies the Lipschitz continuity: $||\nabla f(x)-\nabla f(y)||\le L(\nabla f)||x-y||$.

$$f:\,{R}^{P}\to R,$$

**Proof**: Let $\nabla f(b)=(2{A}^{T}A+\frac{2}{{n}_{1}}{D}_{1}+\frac{2}{{n}_{2}}{D}_{2})b-2{A}^{T}Y$, where D1 and D2 are p*p diagonal matrices. The j-th diagonal element in D1 is ${\Vert {C}_{1}^{(j)}\Vert }_{2}^{2}$, i.e., the 2-norm square of the j-th column in the matrix C^1^. Similarly, the j-th diagonal element in the D_2_ is ${\Vert {C}_{2}^{(j)}\Vert }_{2}^{2}$, and f(b) is the 2-norm of b. Thus, f(b) is a smooth convex function.

Since $\nabla f(x)-\nabla f(y)=(2{A}^{T}A+\frac{2}{{n}_{1}}{D}_{1}+\frac{2}{{n}_{2}}{D}_{2})(x-y)$, we have $||\nabla f(x)-\nabla f(y)||\le L(\nabla f)||x-y||$ and the minimum Lipschitz constant L(∇f) for the gradient ∇f is ${\lambda }_{\max }(2{A}^{T}A+\frac{2}{{n}_{1}}{D}_{1}+\frac{2}{{n}_{2}}{D}_{2})$.

As a smooth convex optimization problem, problem P can be transformed into the following model according to the gradient descent method and References^20,21^.

$$\begin{array}{ccc}\mathop{{\rm{\min }}}\limits_{b}\frac{L}{2}||b-({b}^{\ast }-\frac{1}{L}\nabla f({b}^{\ast })|{|}_{2}^{2}+{\lambda }_{1}||b|{|}_{1}+{\lambda }_{2}\sum _{i,j}|{b}_{i}-{b}_{j}| & L > 0,\,b\ast \,{is}\,{given} & ({P}^{\ast })\end{array}$$

By the iterative soft-thresholding algorithm (ISTA), the solution to problem P* can be iterated according to the formula below:

Therefore, according to the ISTA method, the solution to the problem P* can be iterated as follows:

1 $${b}_{k+1}=\mathop{\min }\limits_{b}\frac{L}{2}||b-({b}_{k}-\frac{1}{L}\nabla f({b}_{k})|{|}_{2}^{2}+{\lambda }_{1}||b|{|}_{1}+{\lambda }_{2}\sum _{i,j}|{b}_{i}-{b}_{j}|\,$$

Thus, the problem boils down to looking for the optimal solution of problem P*, which can be denoted as ${\hat{b}}_{{\lambda }_{1}}^{{\lambda }_{2}}$. Next, the lemma in References^22,23^ can be introduced.

**Lemma 2**. L1 norm soft-thresholding method: For any ${\lambda }_{1},{\lambda }_{2}\ge 0\,$, the optimal solution to problem P* is ${\hat{b}}_{{\lambda }_{1}}^{{\lambda }_{2}}=\mathrm{sgn}({\hat{b}}_{0}^{{\lambda }_{2}})\circ \,\max ({\hat{b}}_{0}^{{\lambda }_{2}}-{\lambda }_{1},0)$, where “o” is the element-wise product operation.

The proof is detailed in the two references.

Since λ2 = 0, the problem (*) can be converted to problem P1 below:

$$\begin{array}{ccc}\mathop{{\rm{\min }}}\limits_{b}\frac{L}{2}||b-({b}^{\ast }-\frac{1}{L}\nabla f({b}^{\ast })|{|}_{2}^{2}+{\lambda }_{1}||b|{|}_{1} & L > 0,\,{b}^{\ast } & {is}\,{given}\,(P1)\end{array}$$

According to Lemma 2 and formula (1), the general ISTA steps of problem P1 can be expressed as:

$${b}_{k+1}=\mathrm{sgn}(\hat{b})\circ \,\max (\hat{b}-{\lambda }_{1},0)$$

$$\begin{array}{cc}\hat{b}={b}_{k}-\frac{1}{L}\nabla f({b}_{k}) & L > 0\end{array}$$

$$\nabla f({b}_{k})=(2{A}^{T}A+\frac{2}{{n}_{1}}{D}_{1}+\frac{2}{{n}_{2}}{D}_{2}){b}_{k}-2{A}^{T}Y$$

The algorithm can be summarized as the following steps:

Input: L = L(∇f), i.e., the Lipschitz constant of ∇f.

(1) Initialize vector b_0_;

(2) Calculate $$\nabla f({b}_{k})=(2{A}^{T}A+\frac{2}{{n}_{1}}{D}_{1}+\frac{2}{{n}_{2}}{D}_{2}){b}_{k}-2{A}^{T}Y$$;

(3) Calculate $$\hat{b}={b}_{k}-\frac{1}{L}\nabla f({b}_{k})$$;

(4) Calculate $${b}_{k+1}=\mathrm{sgn}(\hat{b})\circ \,\max (\hat{b}-{\lambda }_{1},0)$$;

(5) Repeat Steps 2~4 until convergence.

## Experimental Results

### Influence of direction on GLCM texture parameters

**Figure 3:**
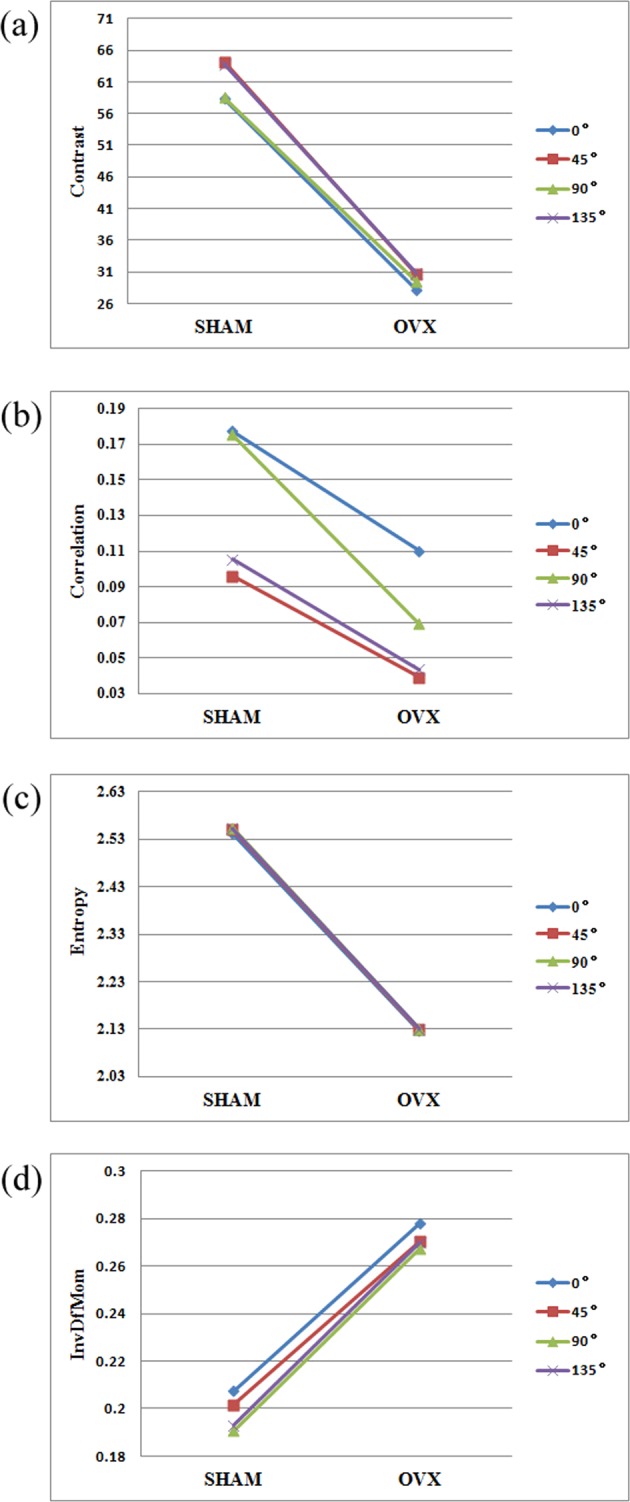
Values of GLCM texture parameters in different directions, (**a**) Contrast; (**b**) Correlation; (**c**) Entropy; (**d**) Inverse difference moment.

The four typical parameters of contrast, correlation, entropy and inverse difference moment were selected to compare the data in the four directions of 0°, 45°, 90° and 135°. Figure 3 shows the values of these parameters at the distance = 3. Specifically, the contrast describes the clarity of the image and the depth of the texture groove; the correlation reflects the similarity of GLCM elements in each direction, and the directionality of texture; the entropy depicts the unevenness or complexity of image texture; inverse difference moment signifies the homogeneity and local variation of image texture.

As shown in Fig. 3, the values of contrast, entropy and inverse difference moment differed slightly in the four directions (the same trend can be observed for the other 7 parameters). However, the correlation was very sensitive to the change of the texture parameters in different directions. Hence, the texture parameters of the GLCM were processed by variation coefficient method.

### Influence of direction on GLRLM texture parameters

**Figure 4:**
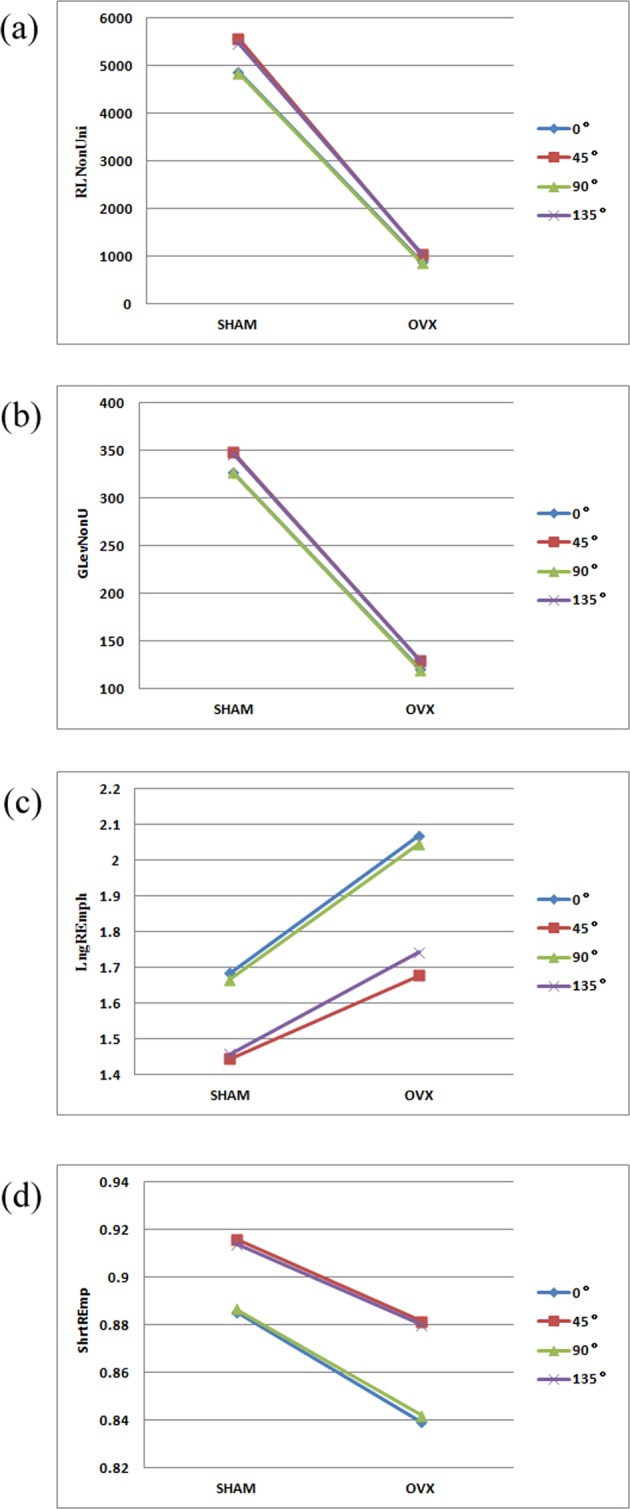
Values of GLRLM texture parameters in different directions, (**a**) Run-length non-uniformity; (**b**) Grey-level non-uniformity; (**c**) Long run emphasis; (**d**) Short run emphasis.

Four parameters, namely, run-length non-uniformity, grey-level non-uniformity, long run emphasis and short run emphasis were selected to compare the data in the four directions of 0°, 45°, 90° and 135° (Fig. 4) below.

As shown in Fig. 4, the values of run-length non-uniformity and the grey-scale non-uniformity differed slightly in the four directions, while those of long run emphasis and short run emphasis were very sensitive to direction. The variation in long run emphasis was greater than that in short run emphasis. Hence, the texture parameters of the GLRLM were processed against the long run emphasis by variation coefficient method.

### Influence of distance on texture parameters

**Figure 5:**
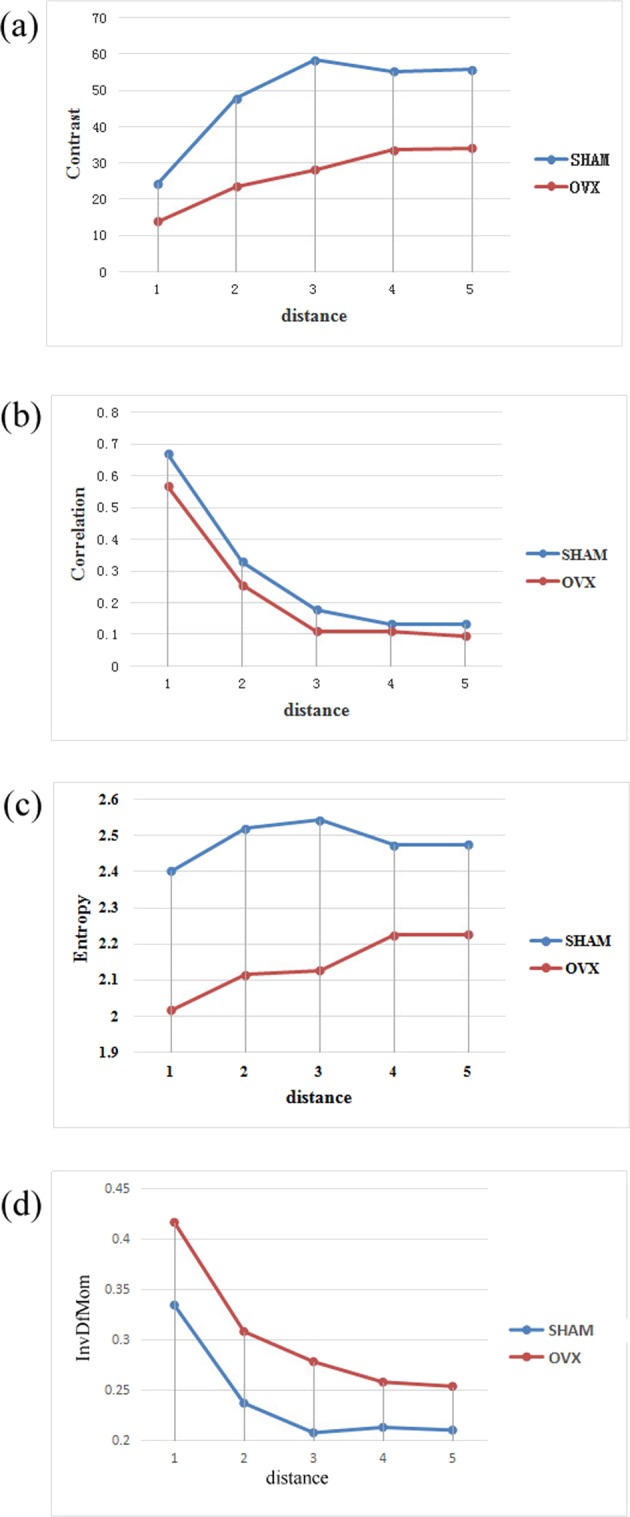
Values of GLCM texture parameters at different distances, (**a**) Contrast; (**b**) Correlation; (**c**) Entropy; (**d**) Inverse difference moment.

The four typical parameters of the GLCM, namely, contrast, correlation, entropy and inverse difference moment, were observed to disclose the variation in parameter value with distances. The variations as the distance increased from 1 to 5 are recorded in Fig. 5 below.

As shown in Fig. 5, the entropy varied less violently than contrast, correlation and inverse difference moment with the increase in distance. Besides, the parameter changes exhibited a gradual declining trend as the distance continued to increase. It can be seen that the values of the four parameters all tended to be stable at the distance of 3 (the same trend can be observed for the other 6 parameters of the GLCM), that is, the robustness was excellent at this distance. In light of this, the distance = 3 was adopted for the weighting process during the extraction of texture parameters from the GLCM.

### Feature selection

Through the proposed convex optimization, 16 parameters were selected out of the 28 shape and texture parameters. The selected parameters include 7 shape parameters (i.e. solidity, shape factor, correction rate, convexity, length-width ratio, invariant moment 3 and invariant moment 5) and 9 texture parameters (i.e. angular second moment, correlation, inverse difference moment, sum average, sum of variance, entropy, difference entropy, grey-level non-uniformity and long run emphasis). In other words, two of the feature parameters of Lasso method were removed, namely, sum of squares and sum entropy.

### Results comparison

It is generally agreed that linear classifiers are good at classifying our data^38,40,41^. Considering the small number of samples, the linear support vector machine (SVM) classifier and the leave-one-out cross validation strategy were adopted to evaluate the classification performance of our method.

The linear SVM classifier was employed to compare the classification results obtained by six different methods: direct averaging the original texture data without using variation coefficient method (TNS); direct averaging the original texture and shape data without using variation coefficient method (TSNS); processing the optimal texture data by variation coefficient method (TYS); processing the optimal texture and shape data by variation coefficient method (TSYS); processing the Lasso-optimized texture and shape data by variation coefficient method (TSYLS); processing the convex optimized texture and shape data by variation coefficient method (TSYLC).

The classification accuracies, sensitivities and specificities of these approaches are listed in Table 1.

**Table 1:** Classification performance of different approaches.

	ACC(%)	SEN(%)	SPE(%)
TNS	68.2	69.32	66.41
TSNS	76.6	77.48	74.28
TYS	77.01	78.2	74.47
TSYS	82.76	84.02	80.22
TSYLS	85.06	86.96	89.13
TSYLC	86.21	88.20	90.22

## Discussion and Conclusions

In this paper, two different image features (i.e. shape parameters and texture parameters) are integrated into an organic whole. Considering the impacts from direction and distance, the texture parameters were optimized for different directions and distances. Then, the parameters were selected by convex optimization method, aiming to reduce the redundancy of feature parameters. The proposed method outperforms the traditional Lasso method and enjoys a good application potential.

### Feature processing

#### Influence of direction on GLCM texture parameters

According to the values of texture parameters in Fig. 3, the SHAM group surpassed the OVX group in both contrast and correlation, indicating that the texture in the former group is relatively clear and uniform. Since the correlation indicates the directionality of the texture, it is learned that both groups carried relatively uniform texture features in the horizontal and vertical directions. This phenomenon is evidenced by the trabecular texture under the high-power lens (Fig. 1). In addition, the SHAM group had a greater entropy than the OVX group. This means the image of the SHAM group outshines that of the OVX group in information quantity, texture complexity and texture uniformity. Furthermore, the inverse difference moment of the SHAM group was smaller than that of the OVX group, revealing that the texture in the former group is more changeable than that in the latter group. These results reflect the decline in BMD and the vagueness of bone structure in the images^42^, and prove that it is feasible to recognize osteoporosis through the texture analysis of trabecular images.

From the variation of each curve in Fig. 3, it can be seen that the values of texture parameters changed in a similar pattern in the four directions, suggesting that the texture parameters can respond to sample texture correctly. In particular, the correlation values fluctuated more significantly than those of the other parameters in the four directions. Hence, correlation was determined as the most direction-sensitive parameter. This agrees well with the concept that correlation can reflect the similarity of GLCM elements in different directions, that is, texture directionality.

#### Influence of direction on GLRLM texture parameters

As can be seen from Fig. 4, the SHAM group surpassed the OVX group in run-length non-uniformity, grey-level non-uniformity and short run emphasis, but lagged behind the latter in long run emphasis. The comparison further proves that the image of the SHAM group features relatively intense texture primitives, complex and fine texture and low grey-level similarity, all of which are the characteristics of osteoporosis images.

In addition, the values of texture parameters changed in a similar pattern in the four directions according to the variation of each curve in Fig. 4. (The trends of long and short run emphases were opposite because the two parameters are antonyms.) The results show that the texture parameters of the GLRLM can respond to sample texture correctly in all directions. Among them, the long run emphasis, an indicator of the number of coarse texture, was the most sensitive parameter to direction.

#### Influence of distance on texture parameters

The values of the texture parameters extracted from the GLCM may change with the distance. Normally, the data at the distance of 1 are selected for further analysis. Nevertheless, when the imaging effect is poor, the number of pixels within the trabeculae is very limited. In this case, the GLCM may be affected greatly by any slight variation in the grey-levels of adjacent pixels. Hence, the selection of distance has a major impact on the results of image analysis. Figure 5 shows that the values of all parameters tended to be stable at the distance of 3. Thus, this distance was selected to calculate the texture parameters of the GLCM.

To disclose the impacts of direction and distance on texture parameters, the parameters must be given different weights in different directions. The variation coefficient method was adopted to calculate the weighting coefficient, thanks to its abilities to highlight the variation in each direction and discriminate between different values. According to Figs 3–5, the weights of the texture parameters of the GLCM were calculated at the distance of 3 by variation coefficient method, and the weights of those in the GLRLM were calculated against the long run emphasis by the same method. After that, the weighted averages of the texture parameters in all directions were derived as the final results.

### Feature selection

As mentioned before, the previous research only extracts a few texture parameters, which eliminates the need for feature selection. In our research, however, a total of 28 shape and texture parameters are extracted, many of which are closely correlated with each other. For example, the correlation coefficient between invariant moments is above 0.8, that between roundness and correction rate stands at 0.97, that between sum entropy and entropy reaches 0.95, and that between run-length non-uniformity and grey-level non-uniformity amounts to 0.92. Meanwhile, both sum entropy and entropy illustrate the complexity of the texture, while the long run emphasis has the opposite meaning of the short run emphasis. To reduce the redundancy of these highly similar parameters, a convex optimization method based on intra-class dispersion was proposed for feature selection. Then, the parameters with nonzero weights were selected for SVM classification. The classification results were compared with those obtained by Lasso-based feature parameters. The results show that our method can achieve a high accuracy with fewer feature parameters.

Among the 16 parameters, the difference entropy had the largest weight coefficient, followed by the sum mean and the convexity. The weight coefficients of invariant moment 3, inverse difference moment, correlation, and entropy were basically the same. These results reveal the importance of the complexity, grey-level variation and structure regularity of trabecular image. As shown in Fig. 1, the SHAM group image indeed had richer and more complex texture than the OVX group image; in terms of shape, the trabeculae in the SHAM group were more regular than those in the OVX group. Hence, the ranking of weight coefficients agrees well with the laws in the images of the two groups.

### Classification performance

It can be seen from Table 1 that the recognition efficiency was not high if only the texture parameters were taken into account, and the accuracy was still 77.01% after the weighting by variation coefficient method. The recognition rate increased obviously after the coupling between texture and shape parameters. The TSNS was 8.4% more accurate than the TNS, and the TSYS was 5.75% more accurate than the TYS. The comparison shows the impacts of texture and shape of trabeculae on classification and recognition. The results are consistent with the shape changes (e.g. thinning and sharpening) of trabeculae in osteoporosis patients.

It can also be learned from Table 1 that the data weighted by variation coefficient method (TYS and TSYS) led to better recognition rate than those not processed by this method (TNS and TSNS). Thus, the impacts of direction on texture should not be overlooked in texture parameter processing, especially when the imaging effect is poor.

Furthermore, the feature selection through the traditional Lasso method (TSYLS) achieved better recognition rate than using all the parameters (TSYS), and the TSYLS exceeded the method without feature selection in accuracy, specificity and sensitivity. However, the proposed method TSYLC outperformed the TSYLS in all indices with fewer parameters. Thus, there is indeed redundancy in the feature parameters, which needs to be eliminated to prevent over-fitting. The proposed method manages to select the most effective parameters for recognition. That is why the TSYLC realized the highest accuracy, specificity and sensitivity.

More importantly, sensitivity and specificity are two leading indices in the clinical application. The two indices directly bear on the rate of missed diagnosis and misdiagnosis. In our experiment, both the TSYLS and TSYLC methods achieved high sensitivity and specificity. The Youden’s indices (YI) of the two methods are:

$${\rm{TSYLS}}:\,{\rm{YI}}=86.96 \% +89.13 \% -1=0.7609$$

$${\rm{TSYLS}}:{\rm{YI}}=88.20 \% +90.22 \% -1=0.7842$$

The greater-than-0.7 YI means the proposed method can be applied to osteoporosis diagnosis, especially when the imaging devices are poor and image features are too vague for artificial diagnosis.

### Further research

The proposed convex optimization method can achieve a high accuracy with a few parameters, despite the low feature dimension. However, the images in our research are 2D microscopic images and only 29 texture and shape parameters are considered. In future, the proposed method will be applied to the analysis of bone densitometry (DXA) or MRI images, and will be integrated with the data on population, blood samples and genes. The purpose is to further improve the diagnosis of bone tissue diseases and the research of bone microarchitecture.

### Ethics approval

This study was carried out in strict accordance with the recommendations in the Guide for the Care and Use of Laboratory Animals of the Guangdong Laboratory Animal Monitoring Institute and the National Laboratory Animal Monitoring Institute of China. The experiments have been conducted according to the protocols approved by Specific Pathogen Free animal care of the Animal Center of Guangdong Medical University, and approved by the Academic Committee on the Ethics of Animal Experiments of the Guangdong Medical University, Zhanjiang, Peoples Republic of China, Permit Number: SYXK (Canton) 2016-0148.

## Data Availability

The datasets generated during and/or analysed during the current study are available from the corresponding author on reasonable request.
